# Increased fear of falling in primary Sjögren’s syndrome: kinesiophobia, impaired balance and poor sleep quality as triggering factors

**DOI:** 10.1007/s00296-025-05847-x

**Published:** 2025-04-15

**Authors:** Serife Seyda Zengin Acemoglu, Ayşegül Özdoğan Bircan, İpek Türk, Ilke Coskun Benlidayi, Sariyildiz Aylin, İlker Ünal

**Affiliations:** 1 Faculty of Medicine, Department of Internal Medicine, Division of Rheumatology, Çukurova University, Adana, Turkey; 2 Faculty of Medicine, Department of Physical Medicine and Rehabilitation, Çukurova University, Adana, Turkey; 3 Faculty of Medicine, Department of Biostatistic, Çukurova University, Adana, Turkey

**Keywords:** Primary Sjögren syndrome, Fear of falling

## Abstract

The aim of this study was to explore the link between fear of falling and factors that may contribute to falls in patients with primary Sjögren’s syndrome (pSS). Our study included a total of 67 patients with pSS, aged between 18 and 65. The following tests were performed on each of them by the same researcher: (I) Pain Quality Assessment Scale (PQAS); (II) Berg Balance Scale (BBS); (III) Pittsburgh Sleep Quality Index (PSQI); (IV) Medical Outcomes Study 36-Item Short Form Health Survey (SF-36); (V) Tampa Kinesiophobia Fatigue Scale Questionnaire (TSK-F); (VI) International Falls Efficiency Scale (FES-I); (VII) Timed Up And Go Test (TUG). Disease activities were recorded using the EULAR Sjögren’s Syndrome Disease Activity Index (ESSDAI) criteria. The following factors were associated with a fear of falling: (I) Deterioration in sleep quality (*p* = 0.031), (II) A decline in general health perception (*p* < 0.001), (III) Increased severity of pain (*p* < 0.001), (IV) Kinesiophobia (*p* < 0.001), (V) Difficulties with timely getting up and walking (*p* < 0.001), (VI) Increased disease activity (*p* < 0.001). There is no information available regarding the prevalence or contributing factors of fear of falling in individuals with pSS. This is the first study to examine fear of falling and contributing factors cross-sectionally in a sample of adults with pSS. These findings could aid in the development of a screening instrument to find modifiable risk factors for individuals with pSS who are susceptible to falling anxiety.

**Supplementary Information:**

The online version contains supplementary material available at 10.1007/s00296-025-05847-x.

## Introduction

Primary Sjögren syndrome (pSS) is a systemic autoimmune disease that primarily causes severe dryness of mucosal surfaces due to damage to the salivary and lacrimal glands. The symptoms of this disease can vary significantly, ranging from mild issues like dry mouth and dry eyes to more serious complications affecting various organs. As a result, pSS can significantly reduce overall quality of life and lead to serious functional limitations [1]. There may be signs of distal sensory and sensorimotor neuropathies, multiple sclerosis-like disorders, encephalitis, aseptic meningitis, cerebellar syndromes, movement disorders, memory, cognition, and depression issues [2]. Furthermore, in patients with pSS, certain studies have demonstrated subclinical vestibular involvement and hearing loss [3]. The ocular, neurological, vestibular, and musculoskeletal systems are just a few of the many systems that play a role in balance. Falls are one of the most dangerous conditions for elderly patients. It is well known that falls cause significant morbidity and mortality in 50% of elderly adults over the age of 80 [4]. 

Fear of falling always causes patients to worry about performing their daily activities effectively. In addition, being anxious about falling can increase the rate of falls with its physical and psychological burden [5]. This concern may increase the risk of injurious falls and may result in activity limitation, decreased quality of life, and increased medication use [6]. Fear of falling is common even in those who have never fallen, despite the fact that falls are associated with the development of this fear [7]. Fall anxiety and falls are not just a problem for the elderly. Research has indicated that patients with fibromyalgia, osteoarthritis, and rheumatoid arthritis may also experience fear of falling and falls [6–10]. 

Fear of falling is a significant risk factor for falls. The aim of this study was to explore the link between this issue and factors that may contribute to falls in patients with pSS.

## Materials and methods

### Participants and study design

The current study was conducted between August 2023 and January 2024; included patients aged between 18 and 64 years and who met the European League Against Rheumatism (EULAR) Sjögren’s syndrome disease activity index (ESSDAI) criteria for pSS [11]. The following exclusion criteria were applied: (I) any other conditions that may affect balance, (II) major organ failure, (III) malignancies, (IV) pregnancy, (V) dementia, (VI) psychotropic drug use, (VII) history of lower extremity/spine surgery or major trauma.

Local Ethics Committee of Faculty of Medicine, Cukurova University granted ethical approval for the study (approval date: 14 July 2023, Number:135/24). Before including patients in the study, the patients gave their written informed consent. The Declaration of Helsinki’s guiding principles were followed when conducting the study.

### Study variables and evaluation methods

Demographic information regarding patients, such as age and gender, was documented. Concurrent laboratory and clinical evaluations were carried out alongside the assessment, or alternatively, verification was obtained from the patients’ medical records. The evaluations included a comprehensive range of tests: the assessment for anti-Ro/SSA and anti-La/SSB antibodies, the examination for antinuclear antibodies (ANA), a labial salivary gland biopsy that indicated focal lymphocytic sialadenitis with a focus score of ≥ 1 per 4 mm², and a Schirmer’s test that demonstrated positivity (≤ 5 mm in 5 min for at least one eye). Researchers attentively observed participants as they engaged in completing study questionnaires in a quiet and conducive environment during face-to-face interactions.

### EULAR Sjögren’s Syndrome Disease Activity Index (ESSDAI)

EULAR has promoted global collaboration by developing disease activity indices for assessing disease activity in patients with pSS. The EULAR Sjögren’s Syndrome Patient Reported Index (ESSPRI) was utilized to evaluate patient-centered measures, including tiredness, dryness, and ache on a scale from 0 to 10. A score of 0 indicates the absence of symptoms, while a score of 10 signifies the most serious symptoms experienced over the past two weeks. The total score is determined by averaging the scores from three different domains. An ESSPRI total score of less than 5 indicates a disease status that the patient finds acceptable, while a score of 5 or higher suggests high disease activity or that the patient is experiencing unsatisfactory symptoms [11, 12]. 

### Pain Quality Assessment Scale (PQAS)

A 20-item questionnaire, called the PQAS, was used to evaluate the quality of pain. The first 19 questions included pain intensity ratings ranging from 1 to 10, where 1 represents “no pain” and 10 indicates “very intense pain.” The final question assessed the duration of the pain. Patients were asked to evaluate their pain status over the past week while answering all the questions [13]. 

### Berg Balance Scale (BBS)

The BBS consists of 14 questions, each scored from 0 to 4 points. The total possible score ranges from 0 to 56. Scores are categorized as follows: 0–20 indicates a high fall risk, 21–40 indicates a moderate fall risk, and 41–56 indicates a low fall risk [14, 15]. 

### Timed Up and Go Test (TUG)

Patients at risk for falls can be identified with the use of a screening tool called TUG. The assessment involves a participant rising from a seated position, walking a distance of 3 m, turning around, returning to the chair, and then sitting down once more. The time taken to complete the test is recorded. Any of the following items, though, were marked: (I) slow and variable pace, (II) short stepping distance, (III) holding on to the wall, (IV) spinning around like a block, (V) loss of balance, (VI) arm swing short or absent, (VII) shuffling feet, (VIII) not using walking aids properly [16]. If a person takes longer than 12 s to complete this test, they may be at risk of falling.

### Tampa Kinesiophobia Fatigue Scale (TSK-F)

This scale is designed to assess a person’s fear of moving their body due to pain. It includes 17 questions, each rated on a scale from 1 to 4. The total score can range from 17 to 68 points, with higher scores indicating increased kinesiophobia in the individual [17–19]. 

### Medical Outcomes Study 36-Item Short Form Health Survey (SF-36)

The SF-36 is an important tool employed in various contexts, including clinical practice, research, health policy evaluations, and general population surveys, to assess quality of life. Its versatility allows for a comprehensive understanding of health-related quality of life across different settings. The SF-36 is a comprehensive questionnaire that evaluates eight health concepts: (I) limitations in physical activities due to health issues, (II) limitations in social activities resulting from physical or emotional problems, (III) limitations in regular role activities because of physical health issues, (IV) bodily pain, (V) general mental health, including psychological distress and well-being, (VI) limitations in regular role activities due to emotional problems, (VII) vitality, which encompasses energy and fatigue levels, and (VIII) overall perceptions of health [20, 21]. As the score rises, we observe that life quality also rises.

### Pittsburgh Sleep Quality Index (PSQI)

There are several questionnaires that have been thoughtfully developed to evaluate sleep quality in clinical populations. Among these, PSQI stands out as a widely recognized tool. This self-rated questionnaire is designed to assess various aspects of sleep quality and disturbances experienced over the course of one month. The assessment is composed of nineteen items that collectively derive seven component scores. These scores reflect subjective sleep quality, sleep latency, sleep duration, sleep efficiency, sleep disturbances, the utilization of sleeping medications, and daytime dysfunction. The scores from these seven components are summed to produce a single overall score. A total score > 5 on the Pittsburgh Sleep Quality Index (PSQI) suggests the presence of clinically significant poor sleep, which may warrant [22, 23]. 

### **International Falls Efficiency Scale (FES-I)**

FES-I assesses an individual’s confidence in performing daily activities without falling. This scale demonstrates adequate reliability, correlates with measurements of balance and gait, and predicts future falls [24–26]. The assessment consists of a total of 16 questions, with scores ranging from 16 to 64. The FES-I scale divides the total scores into three categories based on the level of fear of falling: low (*score: 16–19*), medium (*score: 20–27*), and high (*score: 28–64*) [24]. 

### Statistical analysis

Categorical variables were presented as counts along with their corresponding percentages, providing a clear overview of the data distribution. In contrast, continuous variables were meticulously summarized using both the mean and standard deviation to capture the average and variability of the data. Additionally, the median and minimum-maximum were included when appropriate, offering further insights into the central tendency and spread of the data. To compare categorical variables between the groups, either the Pearson Chi-Square Test or Fisher’s Exact Test was employed, depending on whether the expected value problem arose. The normality of distribution for continuous variables was confirmed with the Shapiro-Wilk test. To compare continuous variables among FES-I groups, a one-way ANOVA or Kruskal-Wallis test was utilized, depending on whether the statistical assumptions were met. To compare continuous variables among FES-I groups, a one-way ANOVA or Kruskal-Wallis test was utilized, depending on whether the statistical assumptions were met. To assess the correlation between measurements, either the Pearson correlation coefficient or the Spearman rank correlation coefficient was utilized, depending on whether the statistical hypotheses were met. A linear regression analysis was performed to identify the most effective predictors of the FES-I score. In the univariate analysis, variables with a significance level of *P* < 0.25 were included in the linear regression analysis. Additionally, an ordinal logistic regression analysis was conducted to determine the significant predictors of the FES-I groups. In the univariate analysis, variables that were significant at the *P* < 0.25 level were included in the logistic regression analysis. All analyses were conducted using IBM SPSS Statistics Version 20.0 (IBM Corp., Armonk, NY, USA). A statistical significance level of 0.05 was used for all tests.

## Results

### Characteristics of the study population

The study included 67 patients (64 female and 3 male) with pSS. The mean age of the patients was 51.9 years. Table 1 provides a summary of demographic information about patients and average results from all surveys.

**Table 1:** Demographic data and study variables of patients with pSS

	Patients (*n* = 67)
**Female sex**, n(%)	64 (%95.5)
**Age** (mean ± SD)	51.9 ± 13.1
**BBS**, median (min-max)	44 (16–52)
**PSQI**, median (min-max)	
Subjective Sleep Quality	1 (0–3)
Sleep Latency	1 (0–3)
Sleep Duration	0 (0–3)
Sleep Efficiency	0 (0–3)
Sleep Disturbance	2 (0–3)
Sleeping Medication	0 (0–3)
Daytime Dysfunction	2 (0–3)
Total PSQI Score (0–21)	7 (1–17)
**TSK-F** (17–68), median (min-max)	42 (29–52)
**SF-36** (0-100), median (min-max)	
Physical Function	45 (5-100)
Physical Role Difficulty	0 (0-100)
Emotional Role Difficulty	100 (0-100)
Energy/Dynamics/Vitality	35 (0–80)
Mental Health	68 (8–92)
Social Functionality	75 (25–100)
Pain	45 (0-100)
General Health Perception	40 (5–85)
**TUG**, median (min-max)	20 (12–38)
**FES-1** (16–64), median (min-max)	25 (16–50)
**ESSDAI**, median (min-max)	2 (0–23)
**PQAS**, median (min-max)	
Pain	6 (0–10)
Sensation of Stinging	3 (0–9)
Feeling of Distress	6 (0–10)
Sense of Coldness	0 (0–9)
Sensibility	3 (0–9)
Sensation of Being Crushed	4 (0–9)
Itchy Pain Sensation	0 (0–7)
Shooting Pain Sensation	3 (0–9)
Sensation of Numbness	2 (0–8)
Feeling of Electricity	0 (0–8)
Sensation of Tingling	0 (0–8)
Cramps In The Body	3 (0–8)
Spread of Pain	4 (0–10)
Feeling of Unhappiness	4 (0–9)
Pain That Throbs	5 (0–9)
An Oppressive Sensation	3 (0–9)
Deep Pain	4 (0–9)
Superficial Pain	2 (0–8)

BBS: Berg Balance Scale, PSQI: Pain Quality Assesment Scala, TSK-F: Tampa Scale Of Kinesiophobia-Fatigue, SF-36: 36-Item Short Form Health Survey, TUG: The Timed Up And Go Test, FES-1: Falls Effıcacy Scale Internatıonal, ESSDAI: EULAR Sjogren Syndrome’s Disease Activity Index, PQAS: Pain Quality Assesment Scale

### Comparative analyses

The comparison of study variables (SF-36, TSK-F, BBS, TUG, PQAS, ESSDAI scores, and ESSDAI stage) among three groups with low, moderate, and high fear of falling is presented in Table 2. Accordingly, as the TSK-F score increased, the fall anxiety also showed an increase (*p* < 0.001). The median ESSDAI score differed significantly among fear of falling groups (*p* = 0.001). All of the patients with low-level fear of falling (*n* = 15) revealed low disease activity. However, based on disease activity stage, there was not any significant difference among groups (*p* = 0.130). Most of the subscales of SF-36 showed a decrease as the level of fear increased.

**Table 2:** Comparative analysis of study variables stratified based on FES-I score

	Falls efficacy scale international (FES-I) (Score: 16–64)			*P*	Effect Size
	Low level fear of falling (*n*:15)	Moderate level fear of falling (*n*:25)	High level fear of falling (*n*:27)		
**36-item short form health survey (SF-36);** median (min-max) (0-100)					
Physical function	80 (60–100)	60 (30–85)	30 (5–75)	**< 0.001**	0.583
Physical role difficulty	100 (0-100)	100 (0-100)	0 (0-100)	**< 0.001**	0.495
Emotional role difficulty	100 (100–100)	100 (0-100)	100 (0-100)	**0.005**	0.160
Energy/dynamics/vitality	70 (15–80)	25 (5–75)	20 (0–65)	**< 0.001**	0.326
Mental health	76 (40–88)	68 (16–92)	60 (8–84)	0.052	0.075
Social functionality	100 (50–100)	75 (50–100)	50 (25–100)	**< 0.001**	0.290
Pain	67.5 (22.5–100)	45 (22–100)	25 (0-100)	**< 0.001**	0.246
General health perception	70 (45–85)	40 (20–75)	20 (5–70)	**< 0.001**	0.499
**TSK-F (17–68);** mean (± SD)	34.67 ± 4.82	41.52 ± 5.02	45.37 ± 4.74	**< 0.001**	0.421
**BBS (0–56)**; median (min-max)	48.6 ± 3.66	45.84 ± 4.39	37.85 ± 7.18	**< 0.001**	0.410
**ESSDAI;** median (min-max)	0 (0–2)	2 (0–12)	2 (0–23)	**0.001**	0.101
**ESSDAI categarization**					
< 5: low stage; n (%)	15 (%100)	20 (%80)	22 (%81.5)	0.130	0.190
5–13: moderate; n (%)	0 (%0)	5 (%20)	4 (%14.8)		
> 14: high; n (%)	0 (%0)	0 (%0)	1 (%3.7)		
**TUG** Total elapsed time (sec); mean (± SD)	15.93 ± 3.081	19.52 ± 3.938	22.41 ± 6.154	**< 0.001**	0.216
Walking slowly and erratically; n (%)	0 (% 0)	0 (% 0)	13 (% 19.4)	**< 0.001**	0.190
Short stride; n (%)	2 (% 13.3)	4 (% 16)	16 (% 59.3)	**0.001**	0,597
Holding on to the wall while walking; n (%)	0 (% 0)	0 (% 0)	3 (% 11.1)	0.125	0.463
He/she turns like mould; n (%)	2 (% 13.3)	6 (% 24)	18 (% 66.7)	**< 0.001**	0.264
Loss of balance; n (%)	0 (% 0)	3 (% 12)	11 (% 40.7)	**0.001**	0.477
Swings their arms sparingly or not at all when walking; n (%)	1 (% 6.7)	0 (%0)	1 (% 3.7)	1.000	0.151
Shuffles her/his feet while walking; n (%)	0 (% 0)	2 (% 8)	9 (% 33.3)	**0.002**	0.384
**PQAS;** median (min-max)					
Pain	2 (0–10)	6 (0–10)	6 (3–10)	**0.007**	0.183
Sensation of Stinging	0 (0–8)	0 (0–8)	5 (0–9)	**0.007**	0.142
Sensation of burning	0 (0–8)	0 (0–7)	3 (0–8)	**0.007**	0.120
Feeling of distress	0 (0–8)	6 (0–9)	7 (0–10)	**0.001**	0.251
Sense of coldness	0 (0–4)	0 (0–8)	1 (0–9)	0.069	0.083
Sensibility	0 (0–7)	4 (0–8)	4 (0–9)	**0.008**	0.128
Sensation of being crushed	0 (0–8)	4 (0–8)	6 (0–9)	**0.013**	0.126
Itchy pain sensation	0 (0–0)	0 (0–6)	0 (0–7)	**0.004**	0.155
Shooting pain sensation	0 (0–7)	4 (0–8)	4 (0–9)	**0.012**	0.127
Sensation of numbness	0 (0–8)	0 (0–8)	2 (0–8)	0.476	0.032
Feeling of electricity	0 (0–8)	0 (0–8)	2 (0–8)	0.412	0.032
Sensation of tingling	0 (0–8)	0 (0–7)	2 (0–7)	0.533	0.017
Cramps in the body	2 (0–8)	3 (0–7)	4 (0–8)	0.114	0.067
Spread of pain	0 (0–6)	4 (0–8)	5 (0–10)	**0.005**	0.163
Feeling of unhappiness	2 (0–10)	6 (0–10)	7 (1–10)	**0.004**	0.196
Pain that throbs	0 (0–8)	5 (0–8)	6 (0–9)	**0.004**	0.119
An oppressive sensation	0 (0–6)	3 (0–5)	3 (0–9)	**0.017**	0.185
Deep pain	2 (0–8)	5 (0–9)	6 (0–9)	**0.006**	0.179
Superficial pain	0 (0–5)	2 (0–7)	3 (0–8)	**0.011**	0.130

BBS: Berg Balance Scale, PSQI: Pain Quality Assesment Scala, TSK-F: Tampa Scale Of Kinesiophobia-Fatigue, SF-36: 36-Item Short Form Health Survey, TUG: The Timed Up And Go Test, FES-1: Falls Effıcacy Scale Internatıonal, ESSDAI: EULAR Sjogren Syndrome’s Disease Activity Index, PQAS: Pain Quality Assesment Scale

The comparison of sleep quality indices (based on PSQI) among fear of falling groups is depicted in Table 3. Level of fear of falling showed a significant increase as the total PSQI score increased (*p* = 0.031). Similarly, a decline in sleep quality, an increase in sleep disturbance and daytime dysfunction were observed in line with an increase in fear level. (*p* = 0.019, *p* < 0.001, and *p* = 0.001, respectively).

**Table 3:** Comparison of FES-1 and PSQI

Pittsburgh Sleep Quality Index (PSQI)	Falls Efficacy Scale International (FES-I) (Score: 16–64)				
	Low level fear of falling (*n*:15)	Moderate level fear of falling (*n*:25)	High level fear of falling (*n*:27)	*P*	Effect Size
*PSQI Components; median (min-max)*					
PSQI sleep quality	1 (1–2)	1 (0–3)	2 (1–3)	0.052	0.085
PSQI sleep onset latency	1 (0–3)	1 (0–3)	1 (0–3)	0.776	0.007
PSQI sleep duration	0 (0–3)	1 (0–2)	1 (0–3)	0.337	0.040
PSQI sleep efficiency	0 (0–1)	0 (0–1)	0 (0–3)	0.215	0.051
PSQI sleep difficulty	1 (0–2)	2 (1–3)	2 (1–3)	**0.003**	0.191
PSQI sleep medication	0 (0–2)	0 (0–3)	0 80 − 3)	0.065	0.075
PSQI daytime dysfunction	0 (0–2)	2 (0–3)	2 (0–3)	**0.005**	0.162
*PSQI Global score; median (min-max)*	4 (1–12)	7 (2–15)	8 (2–17)	**0.031**	0.102

The comparison of TUG indices and median PQAS scores among the fear of falling level groups was provided in Table 2. Accordingly, a decline in most of the TUG indices was observed as the level of fear increased. Similarly, most of the PQAS scores showed an increase in patients with high-level fear of falling.

### Correlation analysis

The results of the correlation analysis between FES-I score and study parameters are given in Table 4. FES-I score showed a statistically significant correlation with PSQI, TSK-F, BSS, SF-36, TUG, ESSDAI and PQAS score.

**Table 4:** The results of the correlation analysis between FES-I score and study parameters

	Falls Efficacy Scale International (FES-I) (Score: 16–64)		
	*P*	Correlation coefficient	95% CI for Correlation coefficient
**Pittsburgh Sleep Quality Index (PSQI)** Subjective sleep quality			
Subjective sleep quality	0.002	0.374	0.148–0.564
Sleep oncet latency	0.886	-0.018	-0.257–0.223
Sleep duration	0.155	0.176	-0.067–0.399
Sleep efficiency	0.047	0.244	0.004–0.457
Sleep disturbance	0.000	0.431	0.213–0.608
Sleeping medication	0.935	0.010	-0.231–0.250
Daytime dysfunction	0.001	0.414	0.193–0.595
Total PSQI score (0–21)	0.012	0.305	0.070–0.508
**Tampa Scale of Kinesiophobia-Fatigue (TSK-F) (17–68)**	< 0.001	0.622	0.449–0.750
**Berg Balance Scale (BBS) (0–56)**	< 0.001	-0.744	-0.835 – -0.613
**36-item Short Form health survey (SF-36)** Physical function	< 0.001	-0.812	-0.880 – -0.710
Physical role difficulty	< 0.001	-0.541	-0.691 – -0.346
Emotional role difficulty	< 0.001	-0.433	-0.610 – -0.215
Energy/dynamics/vitality	< 0.001	-0.531	-0.684 – -0.333
Mental health	0.042	-0.249	-0.462 – -0.009
Social functionality	< 0.001	-0.638	-0.762 – -0.470
Pain	< 0.001	-0.558	-0.704 – -0.367
General Health Perception	< 0.001	-0.671	-0.785 – -0.514
**The Timed Up and Go test (TUG)**	0.000	0.509	0.306–0.668
**ESSDAI/Activation stage**	0.012	0.306	0.071–0.509
**Pain Quality Assesment Scale (PQAS)** Pain	0.001	0.391	0.166–0.577
Sensation of Stinging	< 0.001	0.433	0.215–0.610
Sensation of burning	< 0.001	0.437	0.220–0.613
Feeling of distress	< 0.001	0.464	0.252–0.634
Sense of coldness	0.003	0.359	0.130–0.552
Sensibility	0.001	0.392	0.168–0.578
Sensation of being crushed	0.002	0.370	0.142–0.560
Itchy pain sensation	< 0.001	0.544	0.349–0.694
Shooting pain sensation	0.002	0.372	0.145–0.562
Sensation of numbness	0.009	0.315	0.081–0.516
Feeling of electricity	0.019	0.285	0.048–0.492
Sensation of tingling	0.053	0.238	-0.002–0.452
Cramps in the body	0.010	0.314	0.080–0.515
Spread of pain	< 0.001	0.485	0.277–0.650
Feeling of unhappiness	0.001	0.393	0.169–0.579
Pain that throbs	0.002	0.484	0.276–0.649
An oppressive sensation	0.001	0.367	0.139–0.558
Deep pain	< 0.001	0.426	0.207–0.604
Superficial pain	0.002	0.371	0.144–0.561

Regression analysis;

The results of the regression analysis are given in Table 5. Accordingly, BSS score, TSK-F score, and “use of sleeping medication” component score from the PSQI were identified as significant factors influencing the concern about falling, as measured by the FES-I score (*p* < 0.001, *p* < 0.001, and *p* = 0.024, respectively).

**Table 5:** The results of the regression analysis

Study variables	*P*	Beta	95% CI
BSS	< 0.001	-0.691	-0.900, -0.481
TSK-F	< 0.001	0.493	0.249, 0.737
PSQI – Use of sleeping medication	0.024	-1.630	-3.068, -0.192

R^2^ = 0.654, Adjusted R^2^ = 0.637

## Discussion

The current study revealed that patients with pSS had increased levels of fear of falling. Additionally, several factors including impaired balance, sleep disturbance, kinesiophobia, perceived pain level, quality of life, and disease activity were associated with the concern about falling. Among those, impaired balance, kinesiophobia, and current use of sleep medication appeared as the determinants of fear of falling.

The anxiety about falling can be just as crippling as the actual fall. Reducing daily activities can create a negative cycle that decreases quality of life and increases the risk of falls. Fall anxiety can be seen in people who have never fallen, even though it is expected of those who have experienced falls in the past [7, 28]. It is well known that people with neurological conditions like Parkinson’s disease, rheumatoid arthritis, diabetes, people who have had joint replacements, and people with other similar conditions can all experience fear of falling [23–35]. One independent risk factor for this fear is having fallen at least once, which roughly triples the chance of developing a fear of falling [35]. None of the participants in our study had never fallen before. Nevertheless, they revealed increased level of fear of falling.

There is no information available regarding the prevalence or contributing factors of fear of falling in individuals with pSS. This study is the first to examine this issue and its contributing factors in a cross-sectional sample of adults with pSS. A person with a concern about falling avoids activities they can perform or has low self-esteem [36]. Fear of falling is a significant health concern that medical professionals must consider, as it profoundly affects quality of life [37]. 

In our study, we found that while sleep quality had a significant impact on fear of falling, sleep duration had no discernible effect on it. We found that efficient sleeping, rather than the amount of sleep, is particularly effective in preventing this fear. This result is remarkably impressive. We have concluded that greater emphasis should be placed on parameters that reflect sleep efficiency rather than just the quantity of sleep. For instance, we should consider how many times a person who sleeps for a total of five hours wakes up during that time, as well as how much uninterrupted sleep they can achieve within those five hours. As anticipated and in previous research, we found that overall health perception declined, physical function was restricted, energy and vitality declined, social functionality declined, and pain increased, all of which were associated with an increased risk of fear of falling [38–40]. In a previous study, fear of falling was higher in patients with a history of Parkinson’s disease and high levels of kinesiophobia [41]. In a similar vein, our study found that kinesiophobic patients had an increased level of fear of falling.

An independent risk factor for fear of falling has been found to be longer time to complete the chairstand test, which indicates a decline in physical functioning [35, 40, 42]. We found similar results in our study. In a timed walk-and-get-up test, people who took longer, walked more slowly and erratically, and took shorter strides while walking were more afraid of falling. Exercising is important in ageing population [43]. It is also an essential determinant of management of autoimmune inflammatory rheumatic conditions [44]. In a study conducted by Anne-Gabrielle Mittaz Hager and colleagues, the physical performance of older adults improved with exercise programs designed to reduce the fear of falling [45, 46]. Ma et al. have conducted a significant meta-analysis revealing a clear and positive association between fear of falling and mortality. This finding underscores the urgent need for physicians to take proactive measures in addressing fear of falling among patients [47]. Our regression analysis identified several important factors affecting fear of falling, including the BSS score, TSK-F score, and the sleeping pill use component score. These results provide compelling data for predicting this fear in patients with pSS. Physicians can easily evaluate these factors during patient’s follow-up, which allows them to identify those at high risk of falling. This enables the implementation of appropriate preventive measures.

There are some limitations of the current study. Many of the selected questionnaires are subjective and pose a risk of bias. Quantitative assessment of dynamic balance has not been performed. Yet, we used TUG test as a measure of fall risk.

In conclusion, these findings could aid in the development of a screening instrument to find modifiable risk factors for individuals with pSS who are susceptible to falling anxiety. It is possible to identify patients who may be at risk of falling by incorporating questions about fear of falling and related factors into routine patient assessments. If a person’s risk of falling is suspected, they may be referred to a healthcare provider, especially a physiatrist, or a falls prevention service, if one is offered, so that modifiable risk factors can be addressed.

## Electronic supplementary material

Below is the link to the electronic supplementary material.

Supplementary Material 1

Supplementary Material 2

Supplementary Material 3

Supplementary Material 4

Supplementary Material 5

Supplementary Material 6

## Data Availability

The datasets used and/or analyzed during the current study available from the corresponding author on reasonable request.

## Declarations

### Disclaimer

The authors state that no part of this manuscript, including the text and graphics, are copied or published elsewhere in whole or in part.

### Open data sharing

Data sharing not applicable to this article as no datasets were generated or analyzed during the current study.
